# Sensorineural Hearing Loss and Phosphodiesterase-5 Inhibitors: A Systematic Review

**DOI:** 10.1055/s-0046-1820094

**Published:** 2026-09-15

**Authors:** Peyton Pinkus, Soroush Farsi, William D. Strickland, Lauren Tong, John L. Dornhoffer, Robert A. Saadi

**Affiliations:** 1 Department of Otolaryngology–Head and Neck Surgery, University of Arkansas for Medical Sciences (UAMS), Little Rock, AR, United States; 2 Library, University of Arkansas for Medical Sciences (UAMS), Little Rock, AR, United States

**Keywords:** phosphodiesterase-5 inhibitor, hearing loss

## Abstract

**Introduction:**

Sudden sensorineural hearing loss (SSNHL) can significantly impact quality of life. Recent studies propose a link between the use of phosphodiesterase-5 inhibitors (PDE-5is) and SSNHL emergence.

**Objective:**

To clarify the relationship between PDE-5i use and the risk of SSNHL development.

**Data Synthesis:**

Following the Preferred Reporting Items for Systematic Reviews and Meta-Analyses (PRISMA) statement, two independent reviewers searched the PubMed, Embase, and Web of Science databases for articles up to December 2023 on hearing loss in PDE-5i users. Out of 190 abstracts initially identified, 6 met the inclusion criteria, totaling 54 patients aged between 37 and 62 years. One study involved 4 female participants, while the rest focused on 50 male patients. Sildenafil, vardenafil, and tadalafil were the most commonly used PDE-5is. All patients experienced sensorineural hearing loss, ranging from moderate to profound, with 4 patients (7%) noting tinnitus and 3 patients (5%), vertigo, in addition to the hearing loss. One patient experienced bilateral hearing loss 15 days after initiating sildenafil consumption. Corticosteroid therapy was the exclusive treatment in all cases. The long-term outcomes following the cessation of the medication were available in 7 cases: 4 patients (57%) had no improvement, 2 patients (29%) fully recovered, and 1 patient (14%) had incomplete resolution of the hearing loss.

**Conclusion:**

The current systematic review underscores the importance of monitoring hearing in PDE5i patients and advising them about the potential risk. Further research with larger samples and control groups is needed to establish a definitive causal link.

## Introduction

Sudden sensorineural hearing loss (SSNHL) is most often defined as sensorineural hearing loss ≥ 30dB over at least 3 contiguous audiometric frequencies in a 72-hour timeframe. 1 Many medications have the potential to induce hearing loss by exerting direct toxicity on the inner ear or auditory nerve, resulting in SSNHL. Examples of drugs known to cause this include: anti-infectives (especially aminoglycosides), cancer medications (particularly cisplatin), loop diuretics such as furosemide, and, more recently, phosphodiesterase-5 inhibitors (PDE-5is). 2

Four PDE-5is are currently approved, including sildenafil (Viagra [Pfizer Inc.]: 1998), tadalafil (Cialis [Eli Lilly and Company]: 2003), vardenafil (Levitra [Bayer AG]: 2003), and avanafil (Stendra [Petros Pharmaceuticals]: 2012), which are used primarily for the treatment of erectile dysfunction and pulmonary hypertension. 3 The most common adverse drug reactions reported include headache, flushing, nasal congestion, nasopharyngitis, and dyspepsia. 4 Recently, PDE5is have been linked to adverse effects on hearing, potentially heightening the risk of SSNHL. 5

The effects of PDE-5is in the cochlea are still uncertain, but some studies suggest a link between PDE-5i use and hearing loss, which could be due to the drugs promoting congestion of nasal erectile tissue, raising middle-ear pressure. Additionally, there are concerns that PDE-5is function by blocking cyclic guanosine monophosphate (cGMP) breakdown, leading to its buildup and subsequent induction of gene expression through transcription-factor protein phosphorylation by specific kinases. Such mechanisms have been associated with damage to the cochlear hair cell. 6 7

The current systematic review provides an overview of the potential association between hearing loss and the use of PDE-5is. It is crucial for prescribing physicians to be aware of the possible adverse hearing effects associated with these drugs. Our goal is to systematically analyze the existing literature to uncover any trends in SSNHL progression following the use of this drug class, and to offer recommendations for management strategies and treatment options.

## Review of the Literature

### Data Sources and Search Strategies

A comprehensive search of the PubMed, Embase, and Web of Science databases from inception to December 2023 was conducted in compliance with the Preferred Reporting Items for Systematic Reviews and Meta-Analyses (PRISMA) statement. 8 The search strategy was designed and conducted by an experienced research librarian with input from the study's principal investigator. A combination of Medical Subject Headings (MeSH), other controlled vocabulary, and keywords were used to search for *hearing loss* , *sensorineural hearing loss* , *deafness* , *sildenafil* , *vardenafil* , and *Viagra* . All languages were included in the search algorithm, but only studies written in English studies or those published in full-length international journals with English translation were included for further analysis. The references of all publications were also subjected to review for potential inclusion. Articles with full texts available were screened, and duplicated articles were removed. A qualitative synthesis of the presentation, risk factors, treatment, and long-term outcomes for all of the studies was employed to integrate the study results. The present review was not registered. The level of evidence is V.

### Eligibility Criteria and Quality Assessment

Eligible studies were those that met all of the following inclusion criteria: adult participants aged ≥ 18 years who experienced hearing loss while on PDE5is or who have taken PDE5is within 30 days of experiencing hearing loss, and subjects with no history of hearing loss or any otologic complications that might have led to or exacerbated the hearing loss. The exclusion criteria were abstracts, commentaries, and publications that were deemed outside the scope of the study. Abstracts, commentaries, review articles, and non-original research and studies in which hearing loss was not explicitly associated with PDE5i use were excluded from the search. Two authors (SF and PP) independently assessed the risk of bias for each of the studies included. Disagreements were resolved through consensus. The United States National Institutes of Health (NIH) Quality Assessment Tool was used to assess the quality of case series/case reports. 9 Methodological Index of Nonrandomized Studies (MINORS) criteria were used for the remaining studies.

### Inclusion Criteria and Study Design

**Fig. 1:**
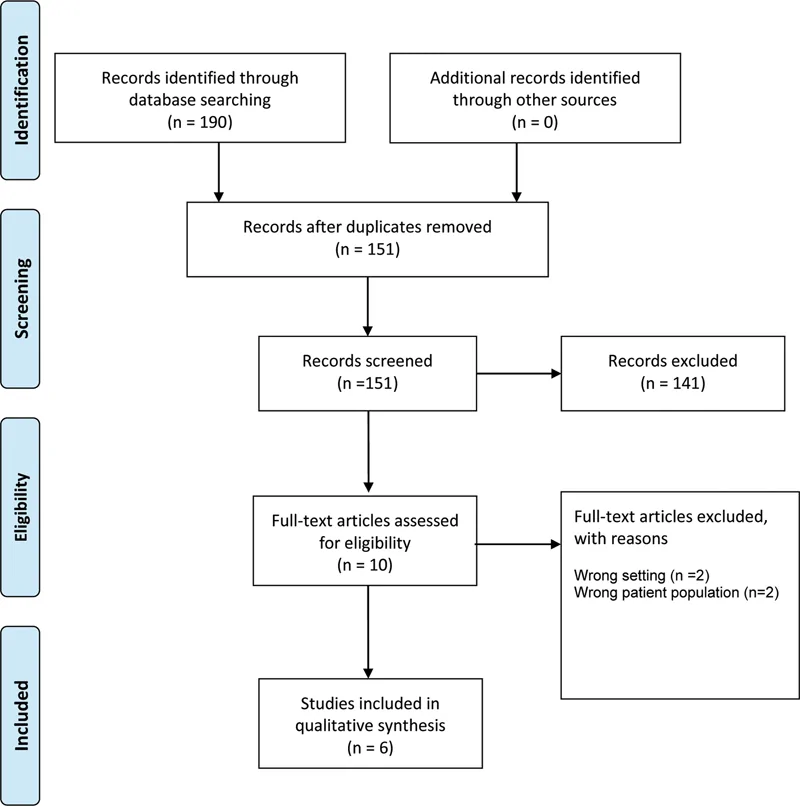
Preferred Reporting Items for Systematic Reviews and Meta-analysis (PRISMA) diagram.

Of the 190 articles initially screened, 39 duplicates were removed, and 151 articles were assessed for eligibility. Of these, 141 were excluded for the following reasons: irrelevant outcomes or populations (such as studies not related to hearing loss or PDE5is), inappropriate study design (such as reviews, commentaries, or editorials), animal or in-vitro studies, and non-English articles without available full-text translations. After screening, ten articles were assessed through full-text screening, six of which met the final inclusion criteria ( Fig. 1 ). Among the included studies, five were case reports, 5 7 10 11 12 and one, a cross-sectional survey 13 ( Table 1 ). Three studies examined populations from North America, 5 10 12 South America, 7 Europe, 13 and Asia. 11 Fig. 1 shows the PRISMA algorithm for article collection and review. In the case of missing data in any of the included studies, it was the authors' intention to avoid bias by reporting only what was explicitly stated in the literature. A statistical analysis was not performed; therefore, the current study lacks statistical biases. Due to variability in study design and outcomes measured, formal synthesis in the form of a meta-analysis was not possible. The following discussion represents a consolidation of the findings on hearing loss experienced by patients taking PDE5i-containing medications.

**Table 1:** Baseline characteristics of the included studies

Study	Publication year	Country	Study type	Patients (N)	Gender (male): N (%)	Mean age (years)	Medication (n)
Barreto and Bahmad 7	2013	Brazil	Case report	2	100	40	Not available
Khan et al. 13	2011	United Kingdom	Retrospective survey-based study	47	91	56.6	Sildenafil (29), tadalafil (10), and vardenafil (7)
Maddox et al. 10	2009	United States	Case report	2	100	58	Case 1: tadalafil; case 2: tadalafil and sildenafil
Mukherjee and Shivakumar 11	2007	India	Case report	1	100	44	Sildenafil
Snodgrass et al. 5	2010	United States	Case report	1	100	57	Vardenafil + non-prescribed erectile dysfunction medication
Wester et al. 12	2018	United States	Case report	1	100	53	Tadalafil

### Risk of Bias within Studies

The detailed results of the quality assessment are provided in. Three studies were rated as presenting fair quality, while two were rated as good according to the NIH Case Report Quality Assessment Guideline. 9 The MINORS criteria score for Khan et al. 13 was moderate, at 10.5.

### Presenting Symptoms

A total of 54 participants were included in the selected studies. Their reported age ranged from 37 to 62 years. Table 1 shows the designs and patient demographics of the studies. Among those included, 1 involved female participants ( *n* = 4), while the remaining studies exclusively focused on male patients ( *n* = 50). The most commonly used PDE-5is were sildenafil, vardenafil, and tadalafil. The time interval between PDE-5i ingestion and the onset of hearing loss was reported for 23 (43%) patients. 5 10 11 12 13 The initial onset of SSNHL occurred within 24 hours of PDE-5i consumption in 21 patients. 5 10 12 13 One of these patients began to experience hearing loss within hours of vardenafil consumption; however, this patient also consumed an additional non-prescription erectile dysfunction medication. 5 Symptoms of hearing loss began 7 days after co-consumption of sildenafil and tadalafil in another patient. 10 In one case report, the patient did not experience hearing loss until day 15 of daily PDE-5i ingestion. A total of 11 patients were reported to experience bilateral hearing loss, while the rest experienced unilateral hearing loss. Four studies reported associated symptoms, such as vertigo ( *n* = 3), 7 10 tinnitus ( *n* = 4), 7 11 12 nausea ( *n* = 1), 10 and aural fullness ( *n* = 1) 12 ( Table 2 ).

**Table 2:** Summary of the reports reviewed

Study	Medication dose	Clinical presentation	Associated symptoms	Imaging	Imaging findings	Treatment	Permanent hearing loss?
Barreto and Bahmad 7	Not available	Sudden hearing loss	Vertigo and tinnitus	Magnetic resonance imaging	Not available	Oral prednisolone 1 mg/kg/day × 10 days + methylprednisolone 0.7 mL intratympanic steroid injection 3x every other day	Yes
Khan et al. 13	Not available	Laterality was recorded in 70% (n = 33): it was unilateral in 88% (n = 29) and bilateral in 22% (n = 4).	Not available	Not available	Not available	Not available	Not available
Maddox et al. 10	Case 1: tadalafil 10 mg; case 2: sildenafil 50 mg and tadalafil 10 mg	Sudden unilateral hearing loss	Case 1: vertigo and nausea	Magnetic resonance imaging of the internal auditory ossicles	Normal	Oral prednisone 1 mg/kg/day × 7 days	Yes
Mukherjee and Shivakumar 11	Sildenafil 50 mg/day × 15 days	Sudden bilateral deafness 15 days after sildenafil consumption	Tinnitus	Not available	Not available	Oral prednisolone 1 mg/kg/day × 1 month + carbogen 8 times/day x 5 days	Yes
Snodgrass et al. 5	Vardenafil 2.5 mg	Sudden right-sided hearing loss	Not available	Not available	Not available	Intravenous dexamethasone 20 mg, followed by intravenous dexamethasone 10 mg every 8 hours × 3 days, followed by oral prednisone taper over 3 weeks starting at 60 mg/day	No
Wester et al. 12	Not available	Right-sided hearing loss, tinnitus, and aural fullness 24 to 48 hours after tadalafil ingestion	Aural fullness and tinnitus	Delayed, intravenous, contrast-enhanced, three-dimensional fluid-attenuated inversion recovery Magnetic resonance imaging	Cochlear hydrops	Oral prednisone × 4 days + nasal steroids (no improvement), acetazolamide 250 mg/day (improvement)	No

### Treatment

Imaging was reported as part of the initial work-up of 5 patients. 7 10 The magnetic reasonance imaging (MRI) scan was conducted on four of these patients. 7 10 Of these four patients, no significant findings were noted for two of them. 10 Imaging findings were not reported for the other two patients. 7 A delayed intravenous, contrast-enhanced, three-dimensional fluid-attenuated inversion recovery (FLAIR) MRI was conducted on the remaining patient, and revealed notable dilation of the right cochlear duct without any hydronic change in the vestibular system. 12 It was suggested that cochlear hydrops was the attributable cause of hearing loss in this patient due to these imaging findings.

Five of the included studies 5 7 10 11 12 reported implemented treatment measures to reverse the PDE5i-induced sensorineural hearing loss experienced by the patients. Each of these studies used corticosteroids for treatment; however, there were differences in dosage, duration, and combination with other medications. The initial treatment included oral corticosteroids in four studies. Two patients were treated with oral prednisolone 1 mg/kg/day for 10 days in one study. 7 In another study, 11 1 patient was also treated with oral prednisolone 1 mg/kg/day; however, their duration of treatment was 1 month. In Maddox et al., 10 2 patients were treated with oral prednisone 1 mg/kg/day for 7 days, and 1 patient received oral prednisone for 4 days. In addition to oral corticosteroids, the initial treatments also included intratympanic methylprednisolone injections 3 times every other day, 7 carbogen 8 times a day for 5 days, 11 and nasal steroids. 12 Due to additional MRI findings of cochlear hydrops in 1 patient, 12 oral corticosteroids and nasal steroids were discontinued, and acetazolamide 250 mg was started. In Snodgrass et al., 5 intravenous dexamethasone followed by oral prednisone was used for treatment.

### Outcomes

Long-term outcomes following cessation of the medication were available in 7 cases: 4 patients (57%) experienced no improvement, 7 10 11 2 patients (29%) fully recovered, 5 12 and 1 patient (14%) 7 had incomplete resolution of the hearing loss. Complete resolution of the hearing loss occurred 4 days after the initiation of intravenous corticosteroid treatment in 1 case report. 5 For another patient, 12 the symptoms of hearing loss were experienced for months after the initial oral and nasal corticosteroid treatment; after additional imaging and the initiation of acetazolamide for treatment, the symptoms fully resolved. Dosage information was inconsistently reported across the included studies. Specific doses were only provided in 3 cases: sildenafil 50 mg/day for 15 days, 11 vardenafil 2.5 mg, 5 and single-use tadalafil 10 mg, 10 with 1 patient consuming both sildenafil 50 mg and tadalafil 10 mg on the same day. 10 The remaining studies did not specify the dose or frequency of PDE5i use, which limits the ability to assess a potential dose-dependent relationship.

The possible causes of PDE-5i-associated hearing loss were provided in two studies. 11 12 Mukherjee and Shivakumar 11 concluded that sildenafil's ototoxic effects caused SNHL due to cochleotoxicity only, as there was no evidence of vestibulotoxicity in the patient. Wester et al. 12 attributed the SNHL following PDE-5i consumption to cochlear hydrops due to the MRI findings. Information on concomitant medication use was not consistently reported across the included studies. In most cases, the patients were described as otherwise healthy, and no details were provided regarding the use of other medications at the time of hearing loss.

## Discussion

Since their introduction to the market, PDE-5is have consistently been among the most prescribed medications annually, 14 and they have long been used in a variety of different medical contexts. The widespread use of this medication class underscores the necessity of vigilance regarding potential adverse effects on different body systems. Recent studies 5 have particularly shown a burgeoning affiliation between PDE-5is and adverse effects on hearing. Such adverse events underscore the drugs' potential serious risk factors, significantly impacting quality of life.

The first case report on the association between sildenafil and SSNHL was published in 2007 in India; 11 the case involved a 44-year-old male patient who experienced bilateral profound sensorineural hearing loss after taking 50 mg/day of sildenafil for 15 days. Consequently, manufacturers were required to include this side effect on their labeling. 7 The first prospective observational study on the effect of PDE5is on hearing was conducted on humans and published in 2009. It involved examining 18 patients undergoing treatment with a PDE5i for erectile dysfunction. Among them, 4 patients demonstrated a statistically significant increase in the hearing threshold in 1 ear, aligning with ototoxicity criteria within 72 hours of drug ingestion. Additionally, all patients displayed a noteworthy unilateral decrease in threshold at 10,000 Hz. However, upon cessation of the drug, the hearing thresholds improved, leading to the disappearance of all instances of hearing loss. 15

Ototoxicity due to PDE-5i has been previously linked to a wide range of symptoms and a variety of presentations. Sudden sensorineural hearing loss can be variable in intensity and frequency, and in the studies 5 7 10 11 12 included in the current reciew, ototoxic effects manifested as vertigo, tinnitus, and cases of hearing loss. A systematic review published in 2019 by Manna et al. 16 reported similar variability among patient presentations. Among the case reports studied in this review, nine patients taking PDE-5is presented with ototoxic symptoms. Specifically, there were two cases of bilateral hearing loss, six cases of unilateral hearing loss, two cases of tinnitus, and three cases of vestibular symptoms such as vertigo and dizziness.

In the present review, most individuals with drug-induced sensorineural hearing loss exhibited unilateral rather than bilateral hearing impairment. 5 10 12 This observation lends support to the notion that unilateral and bilateral SSNHL may represent distinct disease processes, with the unilateral condition being more prevalent and largely idiopathic in nature. 17 Conversely, the bilateral condition is rare and often linked with severe systemic conditions, as noted by Sara et al. 17 Examining any differences in disease mechanisms and progressions between unilateral and bilateral hearing impairment may help concentrate treatment and prevention.

In 2018, Liu et al. 18 described the risk of SSNHL associated with PDE-5i use through a database study. The cohort consisted of 377,722 men using a PDE-5is and 1,957,233 nonusers, all of whom were selected from the MarketScan Commercial Claims and Encounters Database between 1998 and 2007. Drug exposure and SSNHL diagnoses were assessed to determine the risk of ototoxicity that comes with current or recent use of PDE-5is. The results of this study 18 showed that the use of PDE-5i is associated with a small but significantly-increased risk of sensorineural hearing loss.

While reports have acknowledged the association between hearing loss and PDE-5is, the exact mechanism of this interaction is still not completely understood. Some authors 10 haver reported that it could be related to the prolonged effects of cGMP within the cochlea. Barreto and Bahmad 7 reported the results of a histopathological study of the temporal bone; they noted the absence of the organ of Corti and outer hair cells in the majority of the cochlear basal turn, providing an explanation for the associated hearing loss in high frequencies. However, these reasonings still do not account for how the drugs themselves are able to sometimes precipitate SSNHL in certain individuals. There is a need for further research to elucidate the underlying reasons for potential adverse effects.

Regarding treatment, it is widely accepted by otolaryngologists that treatment with corticosteroids is often considered a primary intervention for idiopathic sensorineural hearing loss. However, in some cases, corticosteroid therapy may not yield the desired outcome. Despite the potential benefits, such as limiting inflammation and preventing further damage to ear structures, corticosteroids may fail to restore hearing in certain individuals. Kuhn et al. 19 state that there is a wide range of additional factors that account for the overall effectiveness of this treatment. Recovery may be partial, it may not occur, or it can be complete, and it can be impacted by various factors such as age at onset, severity of hearing loss, presence of vertigo, and time between onset and visit with a treating physician. 19 For several cases in our patient population, treatment with oral corticosteroids was ineffective in restoring hearing, and the patients were left with varying degrees of permanent hearing loss. For these patients, alternative treatment approaches such as hearing aids, cochlear implants, or experimental therapies may be warranted. It is imperative for further research to continue exploring methods to better address the issue of sensorineural hearing loss, particularly when it is idiopathic or due to drug toxicity.

There are several limitations to consider regarding the current systematic review. A significant limitation is the small number of patients with documented long-term hearing outcomes, with follow-up data only available for seven individuals. Given that most of the included reports were single-patient case studies and outcome data were only available for seven patients, no definitive conclusions can be drawn about causality. The findings should be interpreted as preliminary evidence intended to raise clinical awareness rather than establish risk. This restricts the ability to draw robust conclusions regarding the effectiveness of corticosteroid therapy or other interventions for PDE5i-associated hearing loss. Comorbid medical conditions and other potential risk factors for SSNHL were not uniformly reported across the included studies. 5 7 10 11 12 Only one case report 11 explicitly stated the absence of other significant medical conditions in the patient. The remaining studies 5 7 10 12 did not provide detailed medical histories or assess predisposing factors such as cardiovascular disease, diabetes, or noise exposure, all of which are known to increase the risk of SSNHL. This lack of information limits the ability to evaluate whether PDE5i use was an independent risk factor for hearing loss in these patients. Of the 54 included subjects, treatment outcomes were only reported for seven individuals, and none of the included studies specifically reported treatment outcomes for female subjects. Associated symptoms, treatment used, and dosage of the PDE-5i were all categories that may have been reported in some cases, but not others. This made it difficult to directly compare findings across all studies. Further research and a greater sample size are needed to confidently suggest management strategies and therapies for sensorineural hearing loss due to PDE-5i use.

## Final Comments

The present systematic review highlights the importance of informing patients using PDE-5is about the potential risk of developing sudden, and possibly permanent, sensorineural hearing loss.
